# Sleep Promoting Effects of IQP-AO-101: A Double-Blind, Randomized, Placebo-Controlled Exploratory Trial

**DOI:** 10.1155/2019/9178218

**Published:** 2019-05-02

**Authors:** Udo Bongartz, Bee-Kwan Tan, Stephanie Seibt, Gordana Bothe, Ralf Uebelhack, Pee-Win Chong, Natalia Wszelaki

**Affiliations:** ^1^Analyze & Realize GmbH, Weißenseer Weg 111, 10369 Berlin, Germany; ^2^InQpharm Group Sdn Bhd, E-16 Plaza Mont Kiara, 2 Jalan Kiara, 50480 Kuala Lumpur, Malaysia; ^3^Analyze & Realize GmbH, Waldseeweg 6, 13467 Berlin, Germany; ^4^Zaluvida Corporate Sdn Bhd, E-16 Plaza Mont Kiara, 2 Jalan Kiara, 50480 Kuala Lumpur, Malaysia

## Abstract

**Objective:**

The purpose of this study was to explore the clinical benefit and tolerability of IQP-AO-101 in healthy subjects with sleep complaints.

**Methods:**

This double-blind, randomized, placebo-controlled trial involved fifty subjects with sleep complaints. Subjects with a Pittsburgh Sleep Quality Index (PSQI) score between 6 and 15 were randomized to receive either IQP-AO-101 or placebo for 6 weeks, following a run-in period of one week. Sleep parameters were assessed at baseline and after 1, 4, and 6 weeks using the modified Athens Insomnia Scale (mAIS). Subjects were also instructed to wear an activity tracker and keep a sleep diary during the study. Other questionnaires administered were the Frankfurt Attention Inventory (FAIR-2) and the Profile of Mood States (POMS-65). Blood samples for safety laboratory parameters were taken before and at the end of the study.

**Results:**

After 6 weeks, subjects who consumed IQP-AO-101 reported significant improvements in mAIS scores compared with placebo, including mAIS total score (11.76 ± 6.85 vs 4.00 ± 4.80;* p* < 0.001); night parameters composite score (5.20 ± 3.80 vs 2.04 ± 3.16;* p* = 0.001); and day parameters composite score (6.56 ± 4.10 vs 1.96 ± 2.65;* p* < 0.001). All individual parameters (Items 1 to 8) were also significantly improved from baseline after 6 weeks of IQP-AO-101 intake. Analysis of variance with baseline values as covariates showed statistically significant improvements across all individual parameters for IQP-AO-101 when compared to placebo. The measurements using the activity tracker, sleep diary, FAIR-2, and POMS did not reveal any significant differences between groups. No adverse effects related to the intake of IQP-AO-101 were reported. Tolerability was rated as very good by all the subjects and by the investigator for all cases.

**Conclusions:**

In this study, IQP-AO-101 was well tolerated and efficacious for promoting sleep and enhancing daytime performance in subjects with moderate sleep disturbances.

**Clinical Trial Registration:**

This trial is registered with ClinicalTrials.gov, no. NCT03114696.

## 1. Introduction

Sleep disorders, characterised by a frequent difficulty to fall asleep or maintain sleep, are the most prevalent health conditions amongst adults. In Europe, prevalence rates vary between countries from 16.5% in Denmark and Italy to 31.2% in Poland [1], with adults between 45 and 64 years old being the most affected by sleep disorders [2].

Sleep disorders and sleep disturbances affect nightly rest causing distress during daytime and impacting negatively on quality of life and work productivity [3, 4]. Long-term effects of sleep deprivation may not only cause fatigue and decrease cognitive performance, but even increase the risk of type 2 diabetes [5, 6], hypertension [7], cardiovascular disease [8], depression [9] and early mortality [10]. Therefore, getting enough sleep of good quality is important for human health.

Apart from lifestyle changes such as improving sleep habits and creating a sleep-friendly environment, prescription drugs such as benzodiazepines and the “z drugs”, particularly zolpidem, zopiclone and zaleplon are frequently used for sleep disturbances. Both groups enhance the effects of the gamma-aminobutyric acid (GABA) at a site on the GABA_A_receptor. However, for both benzodiazepines and the “z drugs”, extensive evidence for harmful side effects and drug interactions can be found in the literature. Reported side effects include morning drowsiness, impairment of cognition and psychomotor performance, amnesia, hallucinations, and even suicidality [11–14]. Furthermore, the use of these hypnotic drugs may lead to dependency, further limiting their usefulness [11]. For all these reasons, most people prefer to turn to safer treatment options such as herbal products.

The history of plant-derived sleep aids goes back as far as ancient times. Plants such as valerian (*Valeriana officinalis* L.), hops (*Humulus lupulus* L.), passion flower (*Passiflora incarnata* L.), lavender (*Lavandula officinalis *L.) or lemon balm (*Melissa officinalis* L.) have been used by healers to reduce anxiety, induce calmness, and promote sleep. Since medicinal plants are made up of a variety of constituents that exert different beneficial properties that may help to combat sleep problems, different herbs are often combined to achieve synergistic effects. However, solid evidence of their efficacy is lacking for many natural products available on the market. To date, clinical trials in humans provide preliminary evidence for only a few herbal sleep aids, such as valerian, passion flower or lemon balm [15–19].

IQP-AO-101 (Night Coach™, InQpharm) is a proprietary formulation that contains asparagus extract, saffron extract, lemon balm extract, vitamin C, vitamin E and zinc. The formulation is designed to improve restorative sleep and rejuvenate body cells for optimal physical and mental performance. A range of studies support the efficacy of individual ingredients used in the formulation to promote sleep. In clinical trials, asparagus extract was found to decrease fatigue and improve quality of life, stress resistance, and the ability to sleep [20, 21], an effect which may be attributed to its ability to stabilize serum and salivary cortisol levels [22]. A 4-week treatment with saffron extract significantly ameliorated mood, reduced anxiety, and improved stress management in healthy adults [23]. Saffron extract and its constituents, safranal and crocins (crocin and its hydrolysis product crocetin), were shown to affect various neuronal pathways relevant to sleep promotion. In a mouse study, saffron aqueous extract and safranal demonstrated muscle relaxant, anxiolytic, and hypnotic effects that were similar to diazepam, suggesting a mechanism of action mediated by GABA-benzodiazepine receptor complex [24]. Crocins demonstrated anxiolytic effects in a rat study [25] and increased the total time of non-rapid eye movement sleep in mice [26, 27]. They were shown to have an antagonistic effect at the 5-HT receptor site [28], thus improving mood, and activating the opioid sigma (1) receptors [29], which ameliorate anxiety-like behavior in rats [30]. For lemon balm, three clinical trials support its potential use for sleep disorders by reducing stress, depression and anxiety [17–19]. It was shown to decrease serum corticosterone levels in mice [31] and its active constituents, namely, rosmarinic acid and the triterpenoids oleanolic acid and ursolic acid were shown to inhibit gamma-aminobutyric acid catabolism, thus increasing GABA levels [32]. The rationale for the use of vitamins C and E in this formulation is based on their antioxidant properties that may prevent short- and long-term memory impairment induced by sleep deprivation, as reported in animal studies by Mhaidat et al. 2015 and Alzoubi et al. 2012, respectively [33, 34]. Vitamin C has been demonstrated to lower cortisol and products of lipid peroxidation caused by sleep deprivation in rats [35], whereas zinc was reported to inhibit N-methyl-D-aspartate (NMDA) receptor and restore glutamatergic transmission for its antidepressant effects [36, 37]. Additionally, zinc seems to be important for sleep in humans as serum concentration of zinc correlates with the quality and amount of sleep [38].

Therefore, the aim of our study was to investigate the potential of IQP-AO-101 to promote sleep in subjects with sleep disturbances and to evaluate its tolerability.

## 2. Materials and Methods

### 2.1. Participants

Fifty (50) generally healthy, male and female volunteers between 23 and 64 years of age were screened for eligibility to participate in the study. All 50 (25 per group) subjects were successfully enrolled. None of the subjects were excluded from the study. Subjects had to meet inclusion criteria such as suffering from non-organic moderate sleep complaints in the past year, as per clinical assessment by the investigator. Sleeping difficulties were assessed at screening with a 19-item self-report questionnaire, the Pittsburgh Sleep Quality Index (PSQI) [39]. Subjects with a PSQI score between 6 and 15 were enrolled in the study. Further inclusion criteria included Body Mass Index (BMI) in the range of 18.5-29.9 kg/m^2^. Exclusion criteria included hypersensitivity to any of the ingredients, pregnancy or nursing, sleeping less than 5 hours per night on average (self-reported), insomnia, and substantial daily sleepiness as well as any medical condition associated with sleep disorder (such as sleep apnoea, restless legs syndrome, and neurological/psychiatric disorder) or which could interfere with the trial results and/or subject safety, as judged by the investigator. Further exclusion criteria included use of drugs and/or supplements which could interfere with the results of the study (e.g. melatonin, stimulants and benzodiazepines); behavioral intervention for sleep difficulties; any lifestyle factors that might be potentially associated with sleep problems such as excessive caffeine intake, shift work, and long distance traveling, significant stressors such as active grieving; alcohol or drug abuse and participation in another study. Subjects also committed to adhere to the study requirements and female subjects committed to use contraception throughout the study duration. All subjects gave their written informed consent voluntarily. The clinical trial was approved by the ethics committee of Charité – Universitätsmedizin Berlin and was performed in compliance with principles of the World Health Organization Declaration of Helsinki [40] and the EU recommendations for Good Clinical Practice.

### 2.2. Interventions

Eligible subjects entered a 1-week run-in period to assess their ability to adhere to requirements to wear an activity tracker (at least 5 times during the week) and completion of a sleep diary as scheduled. Thereafter, subjects were randomly allocated to either IQP-AO-101 or placebo group at a ratio of 1:1 according to the randomization code provided by an independent statistician. Subjects were instructed to mix one sachet of the investigational product (either IQP-AO-101 or placebo) in a glass of water (100ml) and consume 30 minutes before bedtime, daily for 6 weeks. Each sachet of the IQP-AO-101 contained the following active ingredients: 300 mg aqueous extract from stem of* Asparagus officinalis*, standardized in cyclo-(L-Ile-L-Pro) content, supplied by Amino Up, Japan; 80 mg lemon balm extract (an aqueous extract of* Melissa officinalis L.* leaf standardized to ≥2% rosmarinic acid, supplied by Pharmanager Ingredients, France); 30 mg saffron extract (a hydro-alcoholic extract of red stigma of* Crocus sativus*, standardized to crocins ≥3%, supplied by Green Plant Extracts, France); 60 mg vitamin C (as calcium ascorbate); 30 IU vitamin E (as dl-tocopheryl acetate) and 10 mg zinc (as zinc gluconate). Standard excipients such as filler, coloring, and flavoring were also added. Placebo sachets contained a similar formulation with the active ingredients replaced by excipient. Both verum and placebo powders were identical in appearance and flavour. Additionally, the subjects were asked to maintain habitual diet, level of physical activity and smoking habits throughout the study.

### 2.3. Measurements

**Figure 1:**
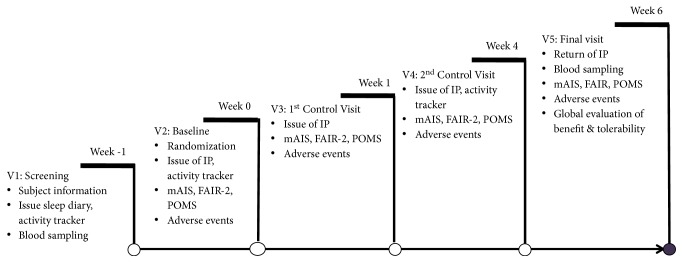
Study schedule.

At each study visit (Figure 1), subjects evaluated the sleep and related daytime parameters by means of the modified Athens Insomnia Scale (mAIS). The Athens Insomnia Scale (AIS) is a validated scale devised to measure subjective estimates of sleep difficulty. It can be used in a large variety of clinical and research settings where the quantification of sleep problems is required [41] The mAIS is made up of 8 items, with four items assessing parameters of nighttime sleep (sleep induction, nighttime awakenings, early final awakening, overall sleep quality) and another four assessing the daytime performance related to sleep (well-being, extent of impact of the sleep problems on daytime sleepiness and functioning during the day, feeling restored/refreshed upon awakening). Each item could be rated from -3 to +3, with -3 corresponding to worst and +3 to best rating.

Additional sleep data was recorded by an activity-tracking device (Fitbit Flex 2, Fitbit, Inc.) during the run-in week, the week preceding visit 3, and the week preceding visit 5. Parameters measured included total sleep time, wake time, number of awakenings, and time in bed. All subjects were also asked to record the time they went to sleep and woke up, along with any stressful events in a sleep diary on a daily basis.

At study visits 2 to 5, subjects also completed the Frankfurt Attention Inventory 2 (FAIR-2) [42] and Profile of Mood States (POMS-65) [43] questionnaires. FAIR-2 measures parameters related to attention quality, global attention performance and continuity in attention. The POMS-65 assesses 6 mood domains, (anger, confusion, depression, fatigue, tension and vigor) using 5-point Likert Scales that range from 0 (not at all) to 4 (extremely) [44]. The Total Mood Disturbance (TMD) was calculated by addition of all other individual scores but subtracting vigor score, and ranged from 0 to 200 points.

Safety assessment included measurement of vital signs (blood pressure and pulse rate) during each visit. Full blood count, clinical chemistry, liver and renal functions, and blood lipid parameters as well as urine analysis were performed at visit 1 and at the end of the study. Adverse events were recorded regardless of causality at every visit.

At the end of the study, subjects and investigator evaluated the benefits and tolerability of the investigational products.

### 2.4. Statistical Methods and Sample Size Determination

The present study was designed as an exploratory pilot study to generate initial data for information regarding benefits and tolerability of IQP-AO-101. The main objective of this study was to evaluate the effect of IQP-AO-101 on the improvement of sleep in healthy subjects experiencing sleep complaints. Therefore, the main endpoint of this pilot study was comparison of changes in mAIS scores from baseline to week 6 between IQP-AO-101 and placebo. Additionally, the effects of IQP-AO-101 on mental performance and mood and the tracker and diary sleep parameters were explored.

The variables were evaluated primarily by non-parametric procedures: Mann-U test for independent groups (comparison of groups) and Wilcoxon test for dependent groups (pre-post comparison within groups). Fisher's exact test was used for comparison of percentages. The influences of baseline values were explored using analysis of variance with baseline values as covariates. Changes in clinical parameters over time (repeated measurements) were analyzed using analysis of variance with respect to differences in groups and systematic changes over time within each group, respectively. All tests were performed with a significance level (type I error) of 5.0% (two-tailed test). The 95% confidence interval was performed. Multiple tests were conducted without correction of significance level in the explorative analysis. Efficacy and safety data were analyzed based on Full Analysis Set (FAS) population. All statistical analyses were done using the SPSS Statistic software, V22.0 (SPSS, Chicago, IL).

## 3. Results

A total of fifty (50) subjects were enrolled in this double-blind, placebo-controlled study between April and July 2017. All subjects completed the study at visit 5. There were no statistically significant differences between the study groups in age, gender distribution, body height, body weight and BMI at baseline. Mean subject age was 44.8 ± 12.7 years, with a higher percentage of female subjects than male subjects in each study group. The baseline characteristics of both study groups are given in Table 1. The PSQI score between the study groups at visit 1 did not differ significantly (*p *= 0.706) indicating that the level of sleep disturbances was similar for both groups at baseline.

**Figure 2:**
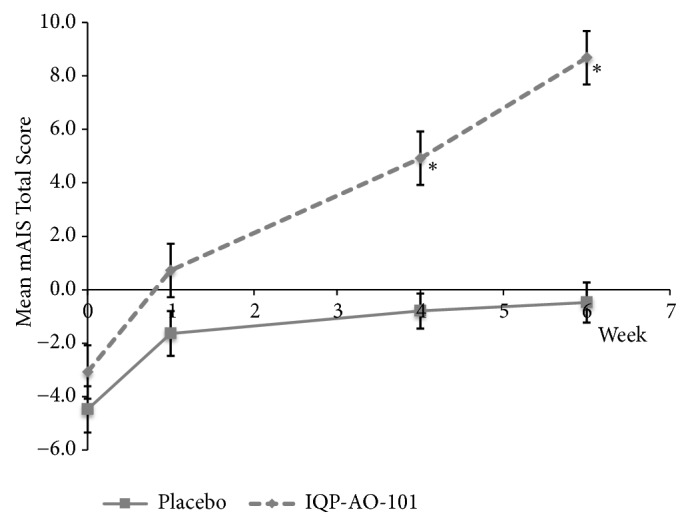
Mean mAIS total score (all parameters) during the 6-week IP intake. *∗p* < 0.001 vs. placebo. Error bars denote standard error of mean.

The verum and placebo groups did not differ significantly at baseline on any of the mAIS parameters measured. However, significant changes were observed after 4 and 6 weeks of IQP-AO-101 intake. The mean increase in mAIS total score was significantly larger from baseline to both week 4 and week 6 in the IQP-AO-101 group than in the placebo group (8.00 ± 7.07 vs. 3.68 ± 4.18,* p* = 0.001 and 11.76 ± 6.85 vs. 4.00 ± 4.80,* p *< 0.001, respectively). The mean mAIS total scores throughout the study are shown in Figure 2.

**Figure 3:**
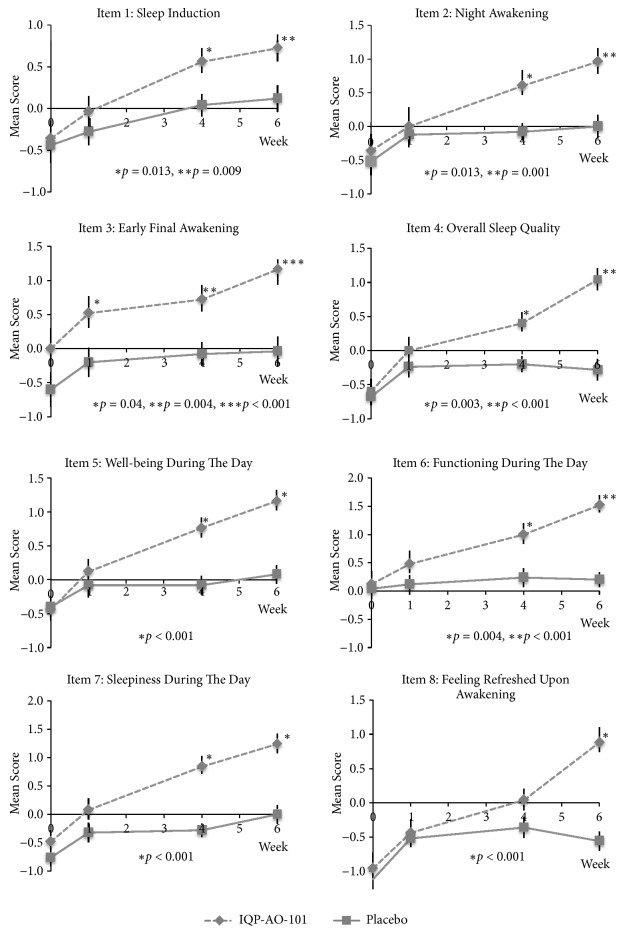
Mean scores of individual mAIS parameters throughout the study. P values for comparison against placebo.

Scores of individual mAIS parameters during the study are shown in Figure 3. There were statistically significant differences between verum and placebo groups at week 6 for all items and at week 4 for Item 1 to 7.

Examining the changes in mAIS scores from baseline to week 6 at the end of the study, significant differences were observed in favor of the IQP-AO-101 group for Items 4-8 when applying the Mann-U test and for all items using the analysis of variance with baseline values as covariates. The differences in the composite scores and the total score were significant in both analyses. These data are presented in Table 2.

However, sleep data obtained with the activity tracking device did not show a significant difference between the study groups with regard to changes in total time in bed, sleep time, wake time and number of awakenings from run-in week (baseline) to the week before v3 (week 1) as well as between baseline and the week before v5 (week 6).Data retrieved from the sleep diary also did not show any significant difference between groups in total time in bed measured during the study.

The FAIR-2 assessment showed no statistically significant differences between the study groups in the attention quality, global attention performance and continuity in attention at any visits.

The POMS-65 assessment showed no significant differences between the study groups in anger, confusion, depression, fatigue, tension and total mood disturbances at any of the visits. A significant difference was reported between groups for factor vigor at visit 3 (verum 18.60 ± 4.90 vs. placebo 15.12 ± 5.26,* p* = 0.025) and 4 (verum 19.84 ± 4.21 vs placebo 16.28 ± 5.15,* p* = 0.015) but not at visit 5.

**Figure 4:**
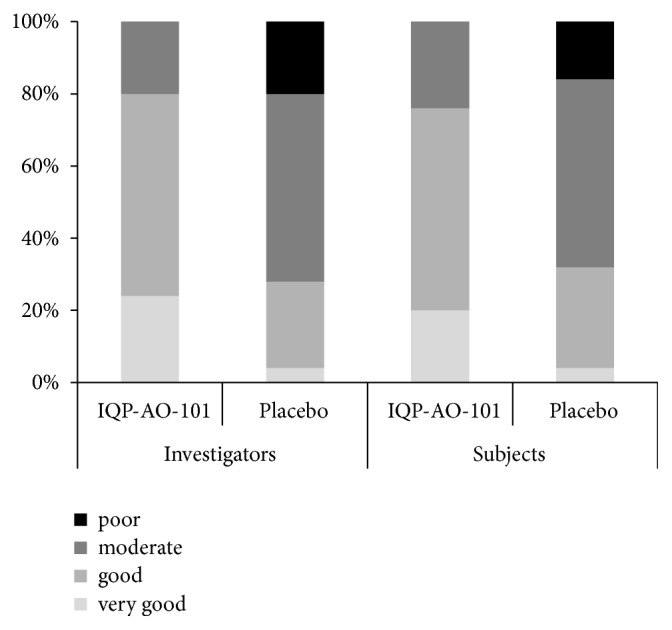
Frequency distribution, global assessment of benefit by investigator and subjects.

At the end of the study, 76% of subjects in the IQP-AO-101 groups rated the benefit of the product as “good” or “very good”, compared to 32% in the placebo group (*p *= 0.007). The benefit assessment by investigator showed similar results (80% vs. 28%,* p *= 0.001) (Figure 4).

A total of 4 adverse events were reported during the course of the study (3 in the verum, 1 in placebo group), including back pain, menstrual cramps, heartburn and common cold. None of the reported adverse events was related to the administration of IQP-AO-101. Additionally, no clinically relevant changes were observed for the safety laboratory parameters (including full blood count, liver and renal functions) at the end of the study for both groups. For IQP-AO-101, all subjects and the investigator rated the tolerability as “very good”.

## 4. Discussion

IQP-AO-101 is a product containing asparagus extract, saffron extract, lemon balm extract, vitamin C, vitamin E and zinc that could potentially alleviate sleep disturbances. It has been developed based on the current scientific literature evidence. The primary objective of this randomized, double-blind, placebo-controlled clinical study was to evaluate safety and efficacy of IQP-AO-101 on sleep parameters over a period of 6 weeks in healthy subjects experiencing sleep disturbances. In the present trial, it was demonstrated that subjects who consumed IQP-AO-101 showed a statistically significant improvement in the mean mAIS total score after 6 weeks. All individual mAIS night parameters related to sleep (sleep induction, night awakening, final awakening and overall quality of sleep) and all parameters related to daytime performance that may be affected by sleep quality (sense of well-being, extent of impact of the sleep problems on daytime sleepiness and functioning and feeling refreshed upon awakening) showed a statistically significant improvement after consumption of IQP-AO-101 for 6 weeks. These findings indicate that IQP-AO-101 may improve nighttime sleep and, hence, as a result improve daytime performance.

The assessment of the sleep data obtained with the activity tracker did not show any significant difference between the IQP-AO-101 or placebo group with regard to tracked total time in bed, sleep time, wake time and number of night awakenings. Similarly, no significant differences between both study groups were observed in total time in bed as reported by subjects in a sleep diary. One explanation for the discrepancy between the findings is the different underlying approach of the mAIS questionnaire and activity tracker to assessing sleep parameters. An activity tracker is a wearable device worn on the wrist that measures every motion caused by wrist or arm. It works based on the assumption that people move most during wake states with a progressive reduction in motion as they approach the deepest stages of sleep, such that sleep/wake states may be discriminated by measuring and analyzing these movements to quantify the sleep patterns [45]. Comparing to polysomnography, the gold standard for measuring sleep, which measures other dimensions of sleep such as brain waves, eye movements, muscle activity, heart physiology and respiratory function in a laboratory setting, activity tracker measured sleep data may suffer in accuracy [46]. On the other hand, mAIS is a subjective assessment which depends on the subjects' retrospective recall on any perceived change in the sleep parameters during the past week. As previously reported, findings for subjective measures do not always agree with objective measures [47]. Nevertheless, subject reported outcomes per questionnaires like mAIS are considered valuable in obtaining the subjects' perspective regarding their treatment experiences.

We investigated the potential effect of IQP-AO-101 on mood states by administering the POMS-65 questionnaire which measures six different dimensions of mood swing, including tension, depression, anger, fatigue, confusion and vigor. However, our results revealed that IQP-AO-101 had not significantly impacted the mood states of the subjects in the current study. This could have been due to a small sample size, large individual variability, and/or generally low level of mood disturbances reported by the subjects at baseline thereby making significant changes difficult to detect.

One important part of this study is that IQP-AO-101 showed an excellent tolerability profile as per evaluation by both participants and investigator. There were no clinically significant changes in subjects' safety parameters and no investigational product-related adverse effects were reported, indicating that the administration of IQP-AO-101 is tolerable for short-term use.

IQP-AO-101 contains ingredients that have previously been shown to alleviate sleep disturbances in animal or human clinical trials. Possible mechanisms of action for this effect may relate to modulation of the hypothalamus-pituitary-adrenal axis, interaction between neurohormonal systems of the brain and protection of neuronal cells from oxidative stress [22, 23, 25, 26, 29, 30, 32, 33, 36–38]. In a study by Ito et al. 2014, asparagus extract was shown to stabilize serum and salivary cortisol levels in humans [22]. Crocin, a saffron extract constituent, was shown to reduce corticosterone level in frontal cortex of stressed rats [48] and in plasma levels of rats exposed to chronic restraint stress [49]. Lemon balm was also observed to decrease serum corticosterone levels in mice [31]. Furthermore, Olayaki et al. 2015 demonstrated recently that vitamin C prevented sleep deprivation-induced elevation in cortisol in the rat plasma [35]. IQP-AO-101 ingredients also seem to be able to modulate several neurohormonal pathways in the brain. Saffron extract and its constituents, safranal and crocins (crocin and its hydrolysis product crocetin), were previously reported to trigger and increase the production of dopamine and glutamate in the brain [50, 51]. In addition, water extract of* Melissa officinalis* L. was reported to modulate serotonergic pathways in rats in a forced swimming test [52]. Furthermore, saffron aqueous extract and safranal showed in a mouse study muscle relaxant, anxiolytic, and hypnotic effects similar to diazepam, suggesting a mechanism of action mediated by GABA-benzodiazepine receptor complex [24]. Active constituents of lemon balm, rosmarinic acid, oleanolic acid and ursolic acid, were shown to inhibit gamma-aminobutyric acid catabolism in an* in vitro* study, thus possibly increasing GABA levels [32]. Yoo et al. 2011 also observed increase in GABA after treatment with lemon balm extract [31]. Zinc might also be involved in the mode of action of IQP-AO-101 as it was observed to inhibit N-methyl-D-aspartate (NMDA) receptor and restore glutamatergic transmission [36, 37]. Neuroprotection due to antioxidative effects could be another mechanism contributing to results observed in this clinical trial. In an* in vitro* cell study by Ogasawara et al. 2014, asparagus extract was shown to scavenge the negative effect of free radicals at the cellular level [53]. In another study, asparagus extract was demonstrated to enhance expression of cytoprotective factors such as heat shock transcription factor 1 (HSF1) and Nrf2 protein [54]. There is also evidence that saffron and its constituents, crocin, crocetin, and safranal can mitigate oxidative stress. In a study on hyperlipidemic rats, both saffron and crocin decreased elevated levels of MDA, glutathione peroxidase enzyme activity (GSHPx), total glutathione (GSH) and oxidized glutathione (GSSG) in serum and increased SOD, CAT, ferric reducing/antioxidant power (FRAP), and total sulfhydryl values in liver tissue with a reduction in thiobarbituric acid reactive species (TBARS) [55]. Moreover, crocin was shown to be a potent antioxidant that combats ischemic stress-induced neuron death by increasing GSH synthesis and thus inhibiting neutral sphingomyelinase activity and ceramide formation. It increased the GSH synthesis via promoting mRNA expression of *γ*-glutamylcysteine synthetase (*γ*-GCS) [56]. In addition, vitamin C was demonstrated to lower products of lipid peroxidation caused by sleep deprivation in a rat model [35]. Further research is needed to elucidate the mechanism of action of IQP-AO-101.

A limitation of this study is that the evaluation of safety and efficacy was performed over a relatively short period of 6 weeks, and no follow-up assessment was performed after IQP-AO-101 had been discontinued. Hence, no information about the benefits on sleep and safety of long-term use is currently available. Furthermore, the study was conducted as a pilot clinical trial without prior estimation of sample size and statistical power, thus it may be difficult to draw firm conclusions from its results. Lastly, it is unknown if the use of IQP-AO-101 would produce similar results in individuals with more pronounced sleep difficulties. Future studies should validate the efficacy of IQP-AO-101 for alleviating sleep problems in a sufficiently powered study with morelarger subject numbers. Its safety and tolerability should also be evaluated over a longer period of time.

## 5. Conclusion and Implication

This is the first trial to investigate the clinical benefit and tolerability of IQP-AO-101 in humans. The clinical study results provide initial evidence that IQP-AO-101 results in improvements in sleep quality, daytime performance, and well-being in subjects with moderate sleep disturbances and is well-tolerated.

## Figures and Tables

**Table 1:** Demographic and baseline characteristic (FAS population). All data are presented as mean ±  s.d.. No significant difference between groups for all variables.

Parameters	IQP-AO-101	Placebo	*P* values
	(*n *= 25)	(*n* = 25)	
Gender:			
Men	9 (36.0%)	6 (24.0%)	0.538
Women	16 (64.0%)	19 (76.0%)	
Age (years)	46.0 ± 12.0	43.5 ± 13.5	0.449
Height (cm)	171.1 ± 7.5	169.4 ± 6.8	0.393
Body weight (kg)	71.6 ± 13.3	67.8 ± 9.8	0.304
BMI (kg/m^2^)	24.3 ± 3.0	23.6 ± 2.5	0.418
Subjects with Anamnestic findings	5 (20.0%)	7 (28.0%)	0.742
Smokers	3 (12.0%)	4 (16.0%)	1.000
PSQI	8.4 ± 2.1	8.4 ± 1.7	0.706

**Table 2:** Overview of mAIS parameter score changes from baseline (v2) to end of study (v5). ^*∗*^*P*_U_ for Mann-U test for independent groups; *P*_group_ for analysis of variance with baseline as covariates, IQP-AO-101 vs. placebo comparison.

mAIS Parameters	IQP-AO-101	Placebo	*P*_U_^ ^^*∗*^ values	*P*_group_^*∗*^ values
	(*n *= 25)	(*n* = 25)		
	v5-v2,	v5-v2,		
	mean ± s.d.	mean ± s.d.		
Item 1: Sleep induction	1.08 ± 1.22	0.56 ± 1.08	0.127	0.012

Item 2: Night awakening	1.32 ± 1.46	0.52 ± 1.36	0.058	0.012

Item 3: Early final awakening	1.16 ± 1.46	0.56 ± 1.23	0.106	< 0.001

Item 4: Overall sleep quality	1.64 ± 1.11	0.40 ± 1.08	<0.001	< 0.001

Night Parameter	5.20 ± 3.80	2.04 ± 3.16	0.001	< 0.001
(Composite score: Item 1-4)				

Item 5: Daytime well-being	1.60 ± 1.12	0.48 ± 1.08	<0.001	< 0.001

Item 6: Daytime functioning	1.40 ± 1.22	0.16 ± 1.11	<0.001	< 0.001

Item 7: Daytime sleepiness	1.72 ± 1.31	0.76 ± 0.88	0.004	< 0.001

Item 8: Feeling refreshed	1.84 ± 1.31	0.56 ± 0.71	<0.001	< 0.001
upon awakening				

Day Parameter	6.56 ± 4.10	1.96 ± 2.65	<0.001	< 0.001
(Composite score: Item 5-8)				

Total score	11.76 ± 6.85	4.00 ± 4.80	<0.001	< 0.001

## Data Availability

No data were used to support this study.
